# Acquired Synergistic Divergence: Contrary to Current Literature

**DOI:** 10.22599/bioj.145

**Published:** 2020-04-22

**Authors:** Martha Waters

**Affiliations:** 1 Manchester Foundation Trust, GB

**Keywords:** synergistic divergence, ocular motility, aberrant regeneration, ephaptic transmission, third nerve palsy, cavernous sinus schwannoma

## Abstract

Current literature reports synergistic divergence as a rare, congenital ocular motility pattern associated with adduction palsy. Its mechanism has been likened to Duane’s syndrome, and some suggest it be referred to as Duane’s Type 4 (Gupta et al. 2010; Schliesser et al. 2016; Wilcox et al. 1981; Khan et al. 2016). There are no published reports of synergistic divergence as an acquired condition, making this case report seemingly the first of its kind. This case report describes an 18-year-old female who presented to clinic in 2013 with symptoms of diplopia and left eye turning outwards. Orthoptic assessment and MRI confirmed a third nerve palsy secondary to cavernous sinus schwannoma. Further monitoring showed progression of the cranial nerve palsy but a stable schwannoma and no aberrant regeneration noted in five years of follow up. The patient was treated with multiple botulinum toxin injections and had squint correction surgery in 2017. Seven months later, synergistic divergence was first noted and remained stable in all following assessments. While the aetiology of acquired synergistic divergence in this case is unclear, we can be confident it is unlikely to be of congenital origin as it was not noted until adulthood and after five years of investigations. This report will discuss possible aetiologies of acquired synergistic divergence and, contrary to current literature, suggest clinicians should consider the possibility that synergistic divergence can be acquired, though is likely to be even rarer than its congenital form.

## Introduction

Synergistic Divergence (SD) is a rare ocular motility disorder associated with congenital adduction palsy (Cruysberg et al. 1959; Mohan et al. 1998; Oystreck et al. 2009; Gupta et al. 2010) and manifests as unilateral divergence of the affected eye on attempted adduction, resulting in bilateral abduction and a “wall-eyed” appearance when looking to the contralateral side.

The earliest report of congenital synergistic divergence is from 1950 (Worth C. and Chavasse F.B.); however, since this time there have been no reports of acquired cases, hence the conclusion in literature to date is that this is a purely congenital condition. The following case report will challenge this conclusion and prompt questions regarding the mechanisms behind acquired SD.

## Presentation, Investigation and Management

**Figure 1:**
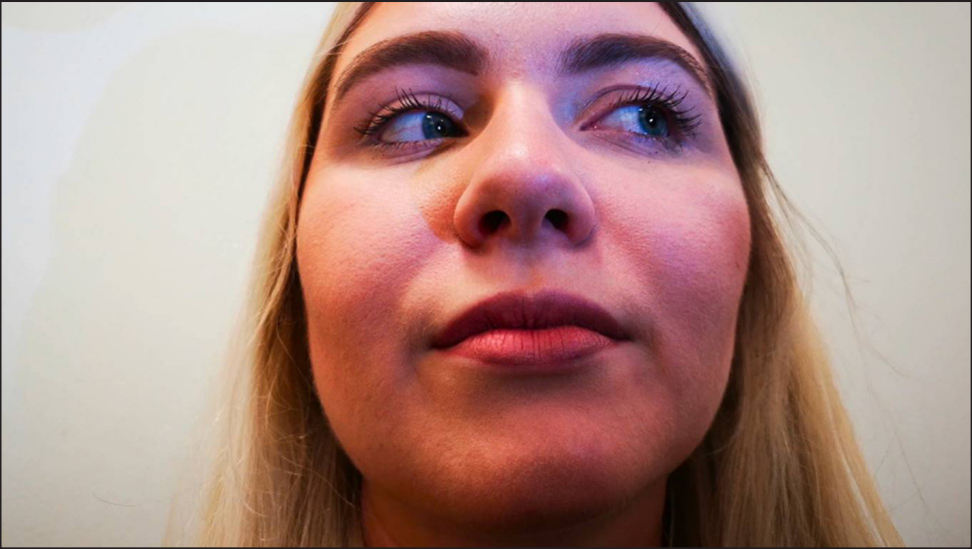
Laevo version. Note the left hypertropia in this position due to the large inferior rectus underaction.

**Figure 2:**
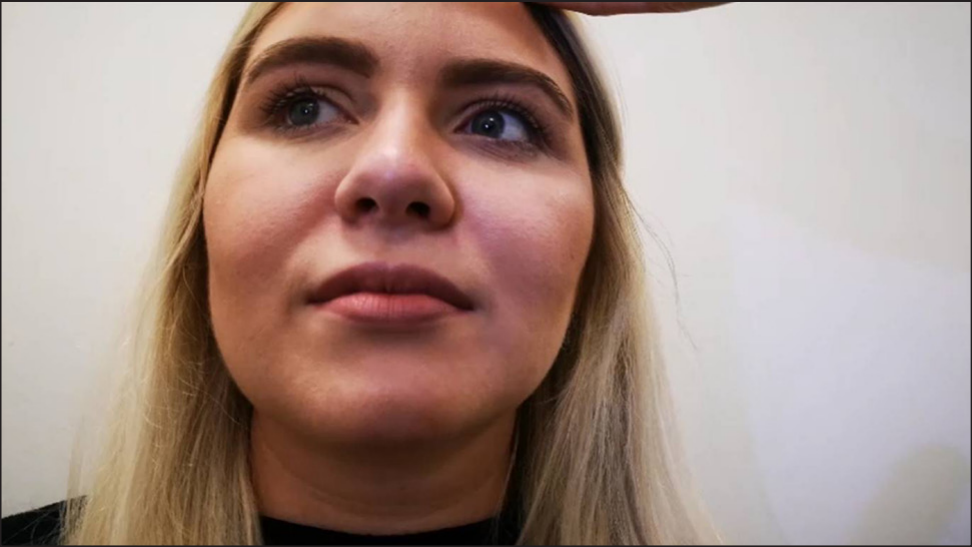
Slight dextroversion.

**Figure 3:**
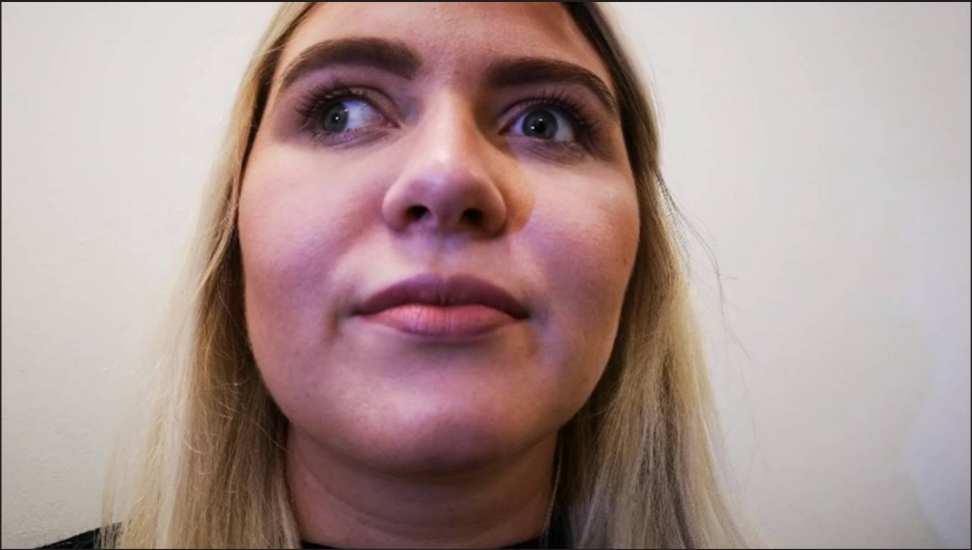
Extreme dextroversion. Note here the left eye has now started to abduct.

An 18-year-old female was referred from her community optometrist with a 2–3 year history of diplopia and her left eye turning outwards, which had been worsening in the last year. Medical history was uneventful, including only tonsillitis and traumatic forceps delivery at birth. Her mother reported the patient had asymmetric pupils, with the left always being bigger and more sluggish, for as long as she could remember. On referral to Manchester Royal Eye Hospital, ocular motility demonstrated a restriction of left adduction and depression, slight left ptosis and anisocoria. Cover test revealed an incomitant exotropia for near and distance with diplopia. Initial diagnosis was a decompensating exophoria; however, this was challenged due to the presence of a –2.5 underaction of the left inferior rectus and sluggish left. In the following 12 months, the restriction of left medial rectus increased to –2 and the initial inferior rectus underaction developed into a restriction with the addition of a –0.5 restriction of left superior rectus. An MRI scan was ordered in November 2013, and this revealed a third nerve schwannoma in the cavernous sinus. The patient was sent for genetic testing to rule out neurofibromatosis type 2 and schwannomatosis. The tests were reported as negative for these conditions. The orthoptic report explicitly stated no aberrant regeneration in 2014. The patient had nine botulinum toxin (BT) injections to the left lateral rectus between 2014 and 2017 to control her diplopia. In 2017, she had a left lateral rectus recession of 5 mm and transposition of the upper muscle border to lower edge of insertion and a left medial rectus resection of 6 mm on adjustable sutures. An inferior oblique transposition was attempted, but a significant torsion effect was observed; therefore, the muscle was returned to its original position. Ocular motility immediately following surgery did not mention any SD or oculosynkenesis. The patient was seen three weeks post operatively by the ophthalmologist who reported the patient had returned to a binocular state, demonstrating an exophoria with good recovery. The patient reported her eye still drifted out when tired, though she could control it relatively well. The patient was then not seen in the orthoptic department until seven months after the surgery, when a new and unusual oculomotor phenomenon was noted. Laevoversion was uneventful, with only the left inferior rectus underaction being notable (Figure 1), however when the patient attempted dextroversion, her left eye would begin to adduct and then briskly abduct, resulting in bilateral abduction (Figures 2 and 3). This was diagnosed as acquired synergistic divergence and assumed to be a rare form of aberrant regeneration. Her symptoms of double vision returned following the development of the SD and had a further BT injection into the left lateral rectus before being given prisms to relieve her diplopia. Currently the patient also uses a slight face turn to the right to aid in her diplopia control.

## Discussion

### Aetiology

This is an unusual case that may be explained by a number of mechanisms. A point of interest in the patient’s history is the sluggish pupil that her mother reported had been present for as long as she could remember. This suggests there may have been pathology associated with the third nerve for many years, possibly since birth. This fits with the diagnosis of a slow-growing schwannoma and explains how she initially presented as a decompensating exophoria in the first instance. It may be that the third nerve palsy had been present since birth and decompensation only happened in adulthood. This theory does not, however, explain the clearly progressive nature of the third nerve palsy in the first 12 months of review and suggests while there may have been pupil involvement for many years, there may not have been any significant extraocular muscle involvement until adulthood.

Synergistic divergence was not reported until 2018, indicating that even if the third nerve palsy had been slowly progressing, the SD does not appear to be longstanding or congenital.

So far there have been no reports of acquired SD in published literature. A majority of papers are in favour of classing congenital SD as an atypical form of Duane’s retraction syndrome (Gupta et al. 2010; Schliesser et al. 2016; Wilcox et al. 1981; Khan et al. 2016), with one electro-oculographic study concluding their two patients with SD demonstrated a close relationship to Duane’s Retraction Syndrome (Cruysberg et al. 1959). This case report suggests SD could also be acquired, but this may be even rarer than its congenital form. Orthoptic reports from 2013 to 2018 were clear in indicating no evidence of aberrant regeneration. Ocular motility was in line with a typical third nerve palsy up until April 2018, when SD was first noted following strabismus surgery. It appears the SD developed post operatively; however, it is difficult to conclude whether it developed as a result of the operation as the patient was not reviewed orthoptically for seven months following surgery. It may be of interest to further investigate the electromyography of the extraocular muscles to understand more about the neural activity in these muscles during attempted dextroversion. Unfortunately, as electromyography was examined prior to surgery, it would not be possible to identify any post-operative changes but may contribute to the understanding of the resulting underlying mechanism of this unusual motility pattern.

The motility pattern was ruled as a rare form of aberrant regeneration at the point of discovery. Incidence of aberrant regeneration following third nerve palsy has been reported at 15% (Chua et al. 2000). It is defined as abnormal regrowth of nerve fibres following damage to the nerve causing co-firing of muscles when the oculomotor nerve is stimulated. In traumatic third nerve palsies, secondary aberrant regeneration will present around 12 weeks after the trauma (Kardon 2000). Primary aberrant regeneration, where there is no nerve palsy demonstrable prior to synkinesis (Weber and Newman 2007) is more indicative of a compressive aetiology and has been reported in cavernous sinus tumours and aneurysms (Schatz et al. 1977; Trobe et al. 1978). The most common presentations of aberrant regeneration are lid retraction on adduction or downgaze, adduction on attempted up or downgaze and pupil constriction on adduction (Weber and Newman 2007; Shuttleworth 1998). The case described above is unusual as it does not quite fit with either of these classifications of aberrant regeneration. Firstly, while the aetiology was compressive, the signs of aberrant regeneration did not present until years after initial presentation. Secondly, synergistic divergence has not been named as a potential form of aberrant regeneration in the literature. This leads us to the following question: can it really be classed as aberrant regeneration?

An alternative explanation to aberrant regeneration is ephaptic transmission: the inter-axonal side-by-side transmission of impulses between adjacent axons, as opposed to typical synaptic neural transmission. This is thought to occur in lesions with a slower growth rate because the slow growing lesion causes damage to the myelin sheath and allows messages to pass between nerve fibre axons, instead of via the synapse (Weber and Newman 2007). This explanation fits more with the case study discussed here, as the diagnosis of schwannoma, long-standing sluggish pupil and deterioration in symptoms later in life indicate a longstanding, slow growing lesion. There is, however, conflicting evidence as to whether or not ephaptic transmission can occur in the third nerve (Sebag and Sadun 1983; Buckley et al. 2005).

The final explanation for synkinesis is central reorganisation. This refers to the degenerative changes to synaptic efficiency unmasking previously unused axonal pathways. This model is not well reported, and some claim it does not consistently explain oculosynkinesis (Sebag and Sadun 1983; Buckley et al. 2005).

### Management

There is limited research into surgical outcome of SD. As a result of acquired SD not being reported before, the studies on surgical correction only include congenital cases and therefore make it difficult to know how well an acquired case of SD would respond to the same procedure.

One study examined surgical outcome of SD in an 8-year-old male who adopted a 40 degree face turn to control his 70 dioptre exotropia (Belanger et al. 2009). He underwent a unilateral lateral rectus recession and medial rectus resection combined with an inferior oblique tenotomy and superior oblique expander, which reduced the head posture by 63% and the exotropia by 83%. The SD significantly reduced post-operatively but did not resolve.

Another study performed a lateral rectus wall fixation and medial rectus recession on 10 patients with SD (Awadein and Zedan 2018). In all cases, the SD resolved and the exotropia was significantly reduced, with all patients measuring less than 10 dioptres base in post-operatively.

While these studies demonstrate a good outcome for those with SD, they have limited sample sizes and all participants have confirmed congenital SD. It is unknown as to whether these surgeries would have as positive outcomes in a case of acquired SD. There may even be contraindication for surgery in acquired cases, as aberrant regeneration (if it is indeed aberrant regeneration after all) may be resistant to surgical improvement and result in a high reoperation rate (Weber and Newman 2007).

## Conclusion

The literature currently only reports synergistic divergence as a congenital phenomenon. The above case report challenges this position as SD was not noted in this particular patient until adulthood, five years after the discovery of a third nerve palsy secondary to a cavernous sinus schwannoma. There is limited literature on the potential mechanisms behind this finding, and while there is some evidence supporting successful surgical outcomes for congenital SD, it is unknown whether they would be as successful in acquired cases. Further research is needed to examine the prevalence of acquired SD and how it can be managed.
